# Binge eating disorders in the age of coronavirus outbreak

**DOI:** 10.1192/j.eurpsy.2021.712

**Published:** 2021-08-13

**Authors:** M. Dhemaid, W. Abbes, S. Hafi, H. Nabli, L. Ghanmi

**Affiliations:** 1 Psychiatry, regional hospital of Gabes, Gabes, Tunisia; 2 Prehospital Emergency Care Service, regional hospital of Gabes, Gabes, Tunisia

**Keywords:** coronavirus outbreak, mental health, General population, Binge eating

## Abstract

**Introduction:**

Lockdown imposed by the Tunisian government had a psychological impact such as depression, stress and anxiety, which triggered the development of eating disorders especially binge eating disorder.

**Objectives:**

To screen the binge eating disorder among general population in Gabes (south of Tunisia) and to identify factors associated with it.

**Methods:**

We conducted a cross-sectional, descriptive and analytical web-based survey, from April 19, 2020, to May 5, 2020 on Facebook on citizens living in south of Tunisia. During this period, the total confirmed cases of COVID-19 exceeded 900 in Tunisia. We used a self-administered anonymous questionnaire containing citizen’s sociodemographic and clinical data. The Diagnostic and Statistical Manual of Mental Disorders (DSM-5) criteria were used to assess Binge-Eating Disorder.

**Results:**

A total of 331 persons were included. They were females (65%) and singles (43,2%). 71% of our population were aged between 20 and 40 years old. Among citizens of southern Tunisia, 6,9% suffered from binge eating disorder during this period of the lockdown. Binge eating disorders were associated to past psychiatric history (2,1% vs 4,53%, p<10^-3^), history of eating disorder (4,5% vs 2,4%, p<10^-3^), social isolation (5,1% vs 1,8%, p=0,015) and lack of physical activity (3,3% vs 3,9%, p=0,025).

**Conclusions:**

Our study showed that lockdown during the COVID-19 pandemic has changed the eating behavior of citizens of southern Tunisia. It is therefore important to screen them in order to manage them before complications emerge.

